# Exercise Intensity and Technical Involvement in U9 Team Handball: Effect of Game Format

**DOI:** 10.3390/ijerph18115663

**Published:** 2021-05-25

**Authors:** Georgios Ermidis, Rasmus C. Ellegard, Vincenzo Rago, Morten B. Randers, Peter Krustrup, Malte N. Larsen

**Affiliations:** 1 Department of Movement and Wellness Sciences, University of Naples “Parthenope”, 80133 Naples, Italy; germidis1990@gmail.com; 2 Department of Sports Science and Clinical Biomechanics, SDU Sport and Health Sciences Cluster (SHSC), University of Southern Denmark, 5230 Odense, Denmark; Rasmuscyril@hotmail.com (R.C.E.); mranders@health.sdu.dk (M.B.R.); pkrustrup@health.sdu.dk (P.K.); 3 Faculty of Health and Sport Sciences, Universidade Europeia, 1500-210 Lisbon, Portugal; vincenzo.rago@universidadeeuropeia.pt; 4 School of Sports Sciences, UiT The Arctic University of Norway, 9037 Tromsø, Norway

**Keywords:** physiology, youth, heart rate, time motion, notational analysis

## Abstract

The purpose of this study was to quantify the exercise intensity and technical involvement of U9 boys’ and girls’ team handball during different game formats, and the differences between genders. Locomotor activity (total distance, distance in speed zones, accelerations, and decelerations), heart rate (HR), and technical involvement (shots, goals, and duels) metrics were collected during various 15 min game formats from a total of 57 Danish U9 players (37 boys and 20 girls). Game formats were a small size pitch (20 × 13 m) with 3 vs. 3 players and offensive goalkeepers (S3 + 1) and 4 vs. 4 players (S4), a medium size pitch (25.8 × 20 m) with 4 vs. 4 (M4) and 5 vs. 5 (M5) players, and a large size pitch (40 × 20 m) with 5 vs. 5 (L5) players. Boys and girls covered a higher total distance (TD) of high-speed running (HSR) and sprinting during L5 games compared to all other game formats (*p* < 0.05; ES = (−0.9 to −2.1), (−1.4 to −2.8), and (−0.9 to −1.3) respectively). Players covered the highest amount of sprinting distance in L5 games compared to all other game formats (*p* < 0.01; ES = 0.8 to 1.4). In all the game formats, players spent from 3.04 to 5.96 min in 180–200 bpm and 0.03 min to 0.85 min in >200 bpm of the total 15 min. In addition, both genders had more shots in S3 + 1 than M5 (*p* < 0.01; ES = 1.0 (0.4; 1.7)) and L5 (*p* < 0.01; ES = 1.1 (0.6; 2.2)). Team handball matches have high heart rates, total distances covered, and high-intensity running distances for U9 boys and girls irrespective of the game format. Locomotor demands appeared to be even higher when playing on larger pitches, whereas the smaller pitch size and fewer players led to elevated technical involvement.

## 1. Introduction

Team handball is an intermittent high-intensity body contact team sport, characterized by sprinting, jumping, throwing, blocking, and pushing [1]. Various studies have described the locomotor demands of team handball in different age and sex groups; on average, international male players cover 4370 ± 702 m [2], elite female players cover 4002 ± 551 m [3], and elite male adolescent players (15 years old) cover 1777 ± 264 m [4]. Overall, the physical and physiological demands, and therefore, the potential as a health-promoting activity of team handball have been predominantly investigated in adults and adolescent players [5]. Only one study investigated U13 boys and girls across different formats [6]. Youth team handball games in Denmark are played on different pitch dimensions and with a different number of players compared to adult games, similar to other team sports such as football [7]. For instance, in youth team handball games, the pitch and goal dimensions are smaller and the number of players is reduced. Extensive research in other team sports showed that manipulating the player numbers and the pitch size can alter the exercise intensity (i.e., locomotor activity, physiological responses) during a game in different sports [8]. Indeed, higher exercise intensity (e.g., heart rate (HR)) is reached when decreasing the number of players and increasing the pitch area [9]. On the other hand, reducing the number of players and pitch dimensions appears to induce higher technical involvement [7,10]. 

Moreover, the sex-specific timing of maturation [11,12] and the gender differences in morphological and neuromuscular characteristics are still early at this stage of age, and gender-related differences in explosive actions are therefore unlikely. Investigating differences in exercise intensity between gender may provide practitioners with a greater understanding of sex-specific training prescription. Overall, several external factors can influence the physiological and technical demands of training drills and thus, the desired conditioning stimulus [13]. Thus, information regarding the exercise intensity in children of both genders across different game formats could be of interest for practitioners involved with youth handball.

Based on previous findings in a study of the game format of U13 handball [6], we hypothesized that a larger court will increase the total distance and that fewer players on the court will increase the involvement of the players in terms of more shots and duels per player.

The purpose of this study was, therefore, to quantify the exercise intensity, the technical involvement, and a gender comparison of U9 boys’ and girls’ team handball during different game formats. The study provides useful knowledge that might change the game format used in tournaments for U9 players for relevant development and health promotion. 

## 2. Materials and Methods

### 2.1. Design

U9 players from ten Danish teams (local handball clubs around the region of Funen) participated in a 1-day tournament. Up to 5 games per player were used. Game formats were classified according to the pitch size and number of players that represent possible official games:

- (S3 + 1): 3 vs. 3 + offensive goalkeepers on a small size pitch size of 20 × 13 m (37 m^2^ per player);
- (S4): 4 vs. 4 on a small size (33 m^2^ per player);
- (M4): 4 vs. 4 on a medium size pitch size of 25.8 × 20 m (65 m^2^ per player);
- (M5): 5 vs. 5 on a medium size (52 m^2^ per player);
- (L5): 5 vs. 5 on a large size pitch size of 40 × 20 m (80 m^2^ per player).

In all of the above formats, goalkeepers participated, but they only were tracked in S3 + 1. To remove the effect of exercise volume and fatigue, the game duration was maintained at 15 min, and the games were played in a randomized order on the same day. All games were played on indoor team handball pitches. The sizes of the goals were 1.6 × 2.4 m on the small pitch, 1.78 × 3 m on the medium pitch, and 2 × 3 m on the large pitch. The games were played with all the official rules of the team handball game. The study was carried out according to the Helsinki protocol.

### 2.2. Participants

Six teams of U9 boys (*n* = 37) and four teams of U9 girls (*n* = 20) participated in the study. All participants were 8–9-year-old recreational handball players.

### 2.3. Activity Profile

The activity patterns were recorded using a wearable device incorporating a 200 Hz accelerometer and gyroscope (Polar Team Pro system, Polar, Kempele, Finland), which was placed on the lower sternum using an elastic band. The following variables were adopted: total distance (TD) covered, peak speed (*V*_peak_) attained, and number of sprints (>18 km/h). Exercise intensity was also distributed in the following running zones: standing/walking (St/W; 0.00–2.99 km/h), jogging (3.00–7.99 km/h), moderate-speed running (MSR, 8.00–12.99 km/h), high-speed running (HSR, 13.00–17.99 km/h), and sprinting (>18 km/h), according to previous studies describing the locomotor demands of team sports’ children [14,15]. 

In addition, the number of accelerations and decelerations were measured with the following zones: Acc < 1.49 m·s^−2^, Acc 1.50 to 2.30 m·s^−2^, Acc > 2.30 m·s^−2^, Dec < −1.49 m·s^−2^, Dec −1.50 to −2.30 m·s^−2^, and Dec < −2.30 m·s^−2^ [14,15]. The total number of accelerations and decelerations was also quantified. The activity profiles and HR data were stored in the device and downloaded using the manufacturer’s software (POLAR, software version 1.3.1, POLAR, Polar Electro Oy, Kempele, Finland) [16].

### 2.4. Heart Rate and Subjective Perceptions

HRs were recorded in 1 s intervals during each game. The HR data were downloaded and expressed as the mean and max HR for the full match. In addition, the HR data were expressed as the time spent in HR zones as follows: <120, 120 to 160, 160 to 180, 180 to 200, and >200 bpm, as previously described [7]. Furthermore, after each game, a Visual Analogue Scale was used to assess the rating of perceived exertion (RPE) and enjoyment/fun (RPF), as previously done in similar studies since it is a well-accepted method to describe subjective phenomena [17,18]. Immediately after the 15 min matches, every player had a paper and pencil to record their scores. All players underwent a brief familiarization session in which three researchers explained the procedure, underlining the importance of scoring their perception of exertion (not fatigue or tiredness). For physical exertion, the players placed a mark on a 17.4 cm line ranging from ‘maximally demanding’ to ‘not demanding at all’, while for perceived fun, a similar line was used, ranging from ‘maximal fun’ to ‘not fun at all’. The result was obtained by measuring with a ruler the length (in centimeters) from 0 to the mark made by the player.

### 2.5. Technical Analysis

Notational analysis was performed by video analysis by five experienced handball coaches (an observer-to-player ratio of 1:1) engaged by the Danish Handball Federation (DHF). The operational definitions of these variables were the following: goal (an attempt with successful scoring), shot (an attempt to score a goal made with any (legal) part of the body, either on or off-target), successful shot (an attempt that successfully scores a goal, given by the ratio between goals and shots and expressed as a percentage), 1 vs. 1 duels (offensive breakthrough to an opponent with the ball) [19,20].

### 2.6. Statistical Analyses

Differences between game formats and between sexes were analyzed using a linear mixed model with unstructured covariance, considering the fact that participants differed regarding the number of game formats they participated in [21]. The game format was set as a fixed effect and the individual subjects and teams were set as random effects. Physical, physiological, and perceptual variables were dependent variables. If a significant effect was found, a pairwise comparison was tested using the Bonferroni post-hoc test. Magnitude-based inferences were adopted to interpret differences between game formats and sexes [22]. Effect sizes (ES) were calculated using mean differences and pooled standard deviation, and classified according to Hopkins and Marshall [22] as following: trivial (ES < 0.2), small (ES = 0.2–0.6), moderate (ES = 0.6–1.2), large (ES = 1.2–2.0), very large (ES = 2.0–4.0), and huge (ES > 4.0). When 90% confidence intervals overlapped positive and negative values, the effect was deemed as unclear. Otherwise, the effect was deemed as the observed magnitude [23]. Significance was set at *p* ≤ 0.05. Data analysis was performed using the Statistical Package for Social Science statistical software (version 23, IBM SPSS Statistics, Chicago, IL, USA) and an online-available Excel spreadsheet [24]. 

## 3. Results

### 3.1. Activity Profile

**Figure 1:**
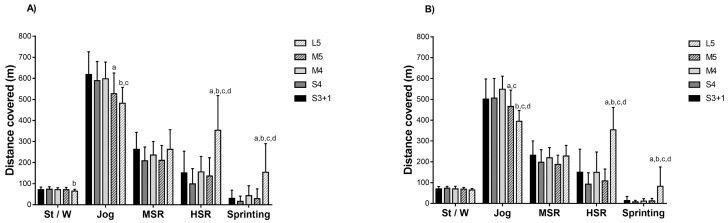
Distance covered in different speed zones in U9 (**A**) boy and (**B**) girl handball players by game formats. S3 + 1: small size, 3 v 3 + offensive goalkeeper; S4: small size, 4 v 4; M4: medium size, 4 v 4; M5: medium size, 5 v 5; L5: large size, 5 v 5. St/W: standing/walking; MSR: moderate-speed running; HSR: high-speed running. ^a^ denotes significant differences compared to S3 + 1; ^b^ to S4; ^c^ to M4; ^d^ to M5 (*p* ≤ 0.05).

Boys covered more TD, HSR, and sprinting and performed more sprints in L5 compared to S3 + 1, S4, M4, and M5 (*p* < 0.05; ES = 0.9 to 1.9). Moreover, the TD was moderately higher in S3 + 1 compared to M5 (*p* = 0.026; ES = 0.9 [0.4; 1.3]). Higher peak speed were reached during L5 compared to S4 and M5 (*p* = 0.01; ES = 0.9 to 1.1) (Table 1) (Figure 1A).

Girls covered more TD during L5 compared to S3 + 1, S4, and M5 (*p* < 0.05; ES = 0.9–2.1). In addition, the TD was moderately higher in M4 compared to M5 (*p* = 0.043; ES = 1.0 [0.4; 1.6]). Higher peak speed were reached during L5 compared to S3 + 1 (*p* = 0.044; ES = 1.0 [1.6; 0.4]). Girls covered more HSR and sprinting and performed more sprints in L5 compared to S3 + 1, S4, M4, and M5 (*p* < 0.05; ES = 0.8 to 2.9) (Table 1) (Figure 1B).

Furthermore, number of Acc_total_ and Dec_total_ were lower in L5 compared to S3 + 1, S4, and M4 (*p* < 0.05; ES = 0.6 to 1.8). In addition, M5 exhibited lower number of Acc_total_ and Dec_total_ than S3 + 1 and S4 (*p* < 0.05; ES = 0.8 to 1.5). The numbers of Acc_<1.5_ and Acc_1.5–2.3_ were lower during L5 compared to S3 + 1, S4, and M4 (*p* < 0.05; ES = 0.7 to 1.6). Conversely, the number of Acc_<1.5_ was moderately higher in S4 compared to M5 (*p* = 0.033; ES = 0.8 (0.3; 1.3)), and the number of Acc_1.5–2.3_ was largely higher in S3 + 1 than M5 (*p* < 0.01; ES = 1.4 (0.8; 1.9)). Notably, lower number of decelerations were observed during L5 compared to S3 + 1, S4, and M4 (*p* < 0.05; ES = 1.0 to 1.3). Furthermore, higher number of Dec_1.5–2.3_, were observed in S3 + 1 compared to M5 and L5 (*p* < 0.05; ES = 0.9 to 1.3). Additionally, S4 showed higher number of Dec_1.5–2.3_ than M5 (*p* = 0.042; ES = 0.9 (0.4; 1.3)). S3 + 1 showed higher number of Dec_>2.3_ than M5 (*p* = 0.019; ES = 0.8 (0.3; 1.3)).

**Figure 2:**
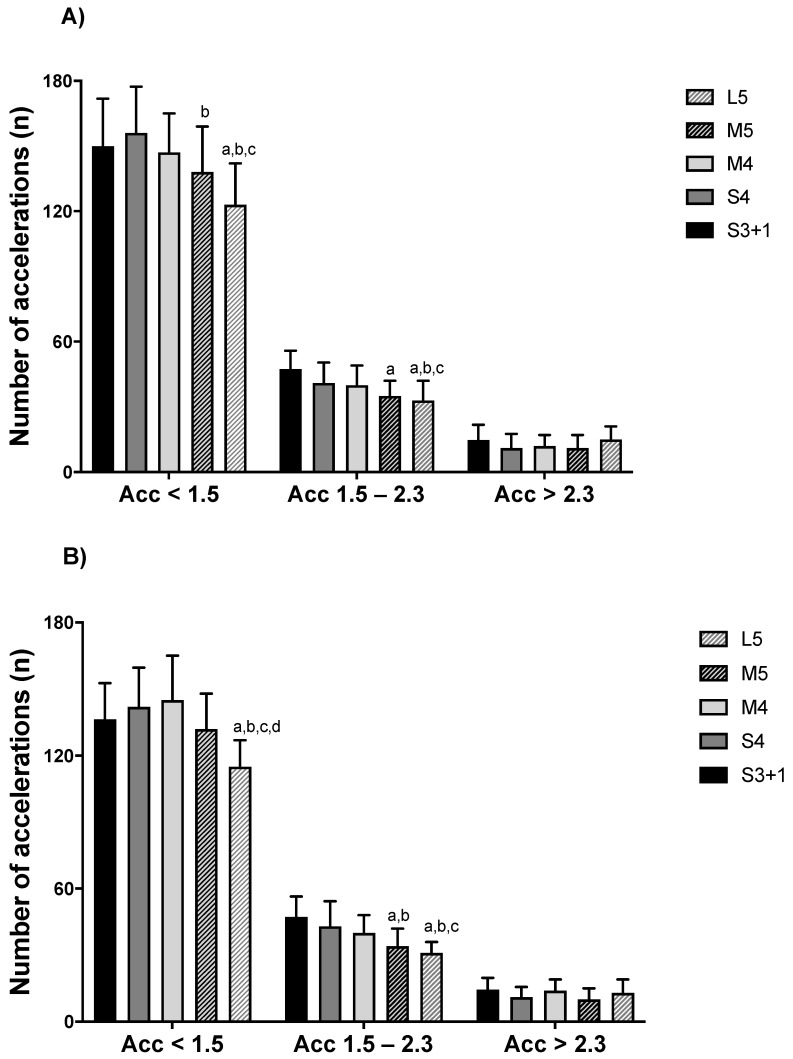
Number of accelerations in U9 (**A**) boy and (**B**) girl handball players by game formats. S3 + 1: small size, 3 v 3 + offensive goalkeeper; S4: small size, 4 v 4; M4: medium size, 4 v 4; M5: medium size, 5 v 5; L5: large size, 5 v 5. ^a^ denotes significant differences compared to S3 + 1; ^b^ to S4; ^c^ to M4; ^d^ to M5 (*p* ≤ 0.05).

**Figure 3:**
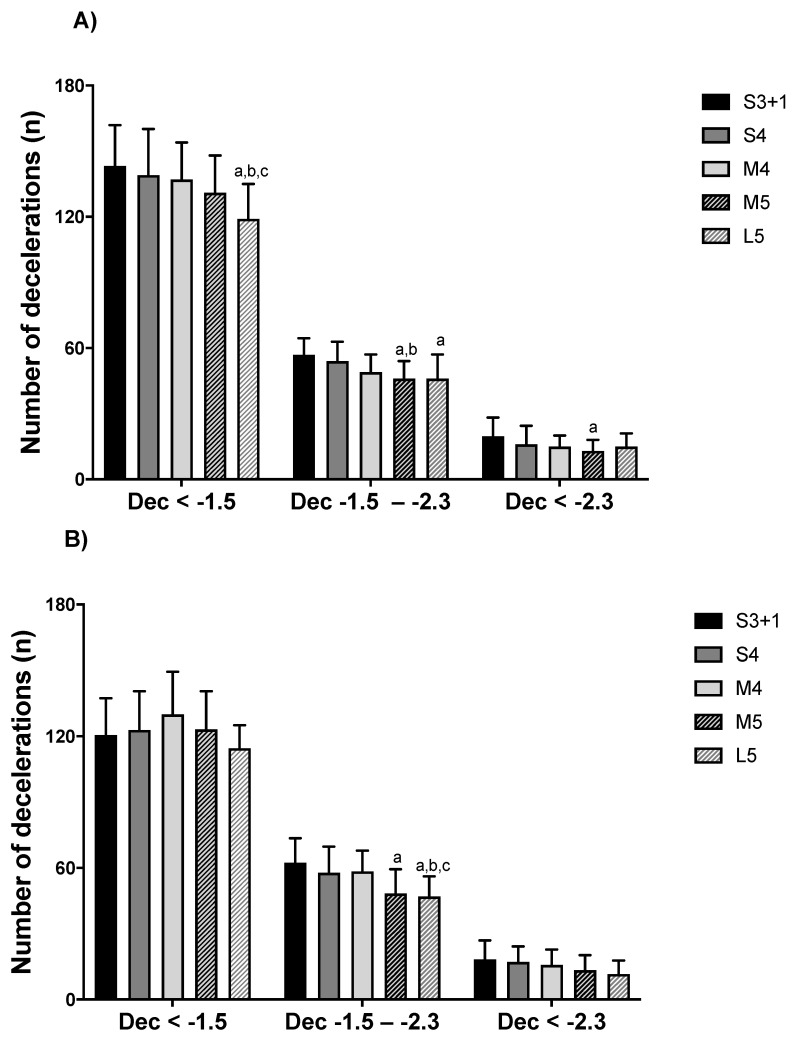
Number of decelerations in U9 (**A**) boy and (**B**) girl handball players by game formats. S3 + 1: small size, 3 v 3 + offensive goalkeeper; S4: small size, 4 v 4; M4: medium size, 4 v 4; M5: medium size, 5 v 5; L5: large size, 5 v 5. ^a^ denotes significant differences compared to S3 + 1; ^b^ to S4; ^c^ to M4 (*p* ≤ 0.05).

For girls, number of Acc_total_ and Dec_total_ were lower in L5 compared to S3 + 1, S4, and M4 (*p* < 0.05; ES = 1.2 to 2.5). In addition, M5 exhibited lower number of Acc_total_ than S3 + 1, S4, and M4 (*p* < 0.05; ES = 0.8 to 1.0). M5 had moderately lower number of Dec_total_ than M4 (*p* = 0.047; ES = 0.9 (0.3; 1.5)). Girls had lower number of Acc_<1.5_ during L5 compared to S3 + 1, S4, M4, and M5 (*p* < 0.05; ES = 1.1 to 1.8). Similarly, during L5, girls had lower number of Acc_1.5–2.3_ than S3 + 1, S4, and M4 (*p* < 0.05; ES = 1.1 to 2.1). In addition, Acc_1.5–2.3_ had fewer efforts in M5 than in S3 + 1 and S4 (*p* < 0.05; ES = 0.8 to 1.4). In Dec_1.5–2.3_, L5 had lower number of efforts than S3 + 1, S4, and M4 (*p* < 0.05; ES = 1.0 to 1.5). In addition, Dec_1.5-2.3_ in S3 + 1 had higher number than M5 (*p* = 0.002; ES = 1.2 [0.6; 1.8]). Detailed representations of accelerations and decelerations are reported in Figure 2 and Figure 3.

### 3.2. Heart Rate and Subjective Perceptions

**Figure 4:**
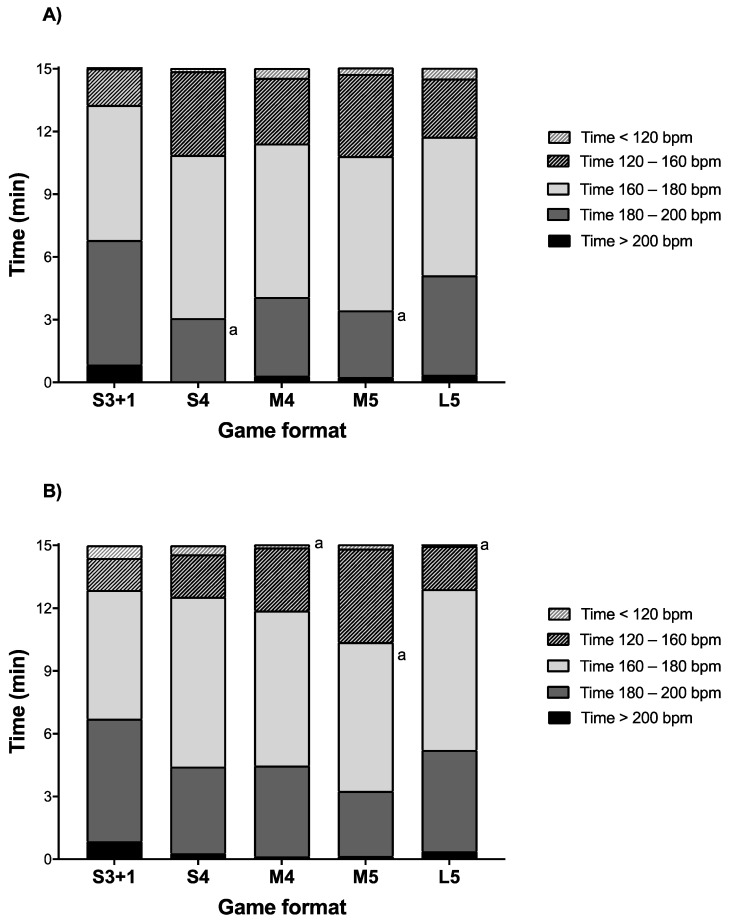
Heart rate distribution during U9 (**A**) boy and (**B**) girl handball games. S3 + 1: small size, 3 v 3 + offensive goalkeeper; S4: small size, 4 v 4; M4: medium size, 4 v 4; M5: medium size, 5 v 5; L5: large size 5 v 5. ^a^ denotes significant differences compared to S3 + 1 (*p* ≤ 0.05).

Boys attained higher HR_peak_ in S3 + 1 compared to S4 (*p* = 0.029; ES = 0.9 (0.4; 1.4)) (Table 1). In addition, boys spent more time within 180–200 bpm in S3 + 1 than in S4 (*p* = 0.045; ES = 0.8 (0.3; 1.3)) (Figure 4). No significant differences were found between game formats in the RPEs and RPFs of boys (*p* > 0.05). Girls had higher times below 120 bpm during S3 + 1 compared to M4 and L5 (*p* < 0.05; ES = 1.3 to 1.6) (Table 1). In addition, girls spent more time between 120–160 bpm in M5 than S3 + 1 (*p* = 0.028; ES = 0.9 (0.3; 1.5)) (Figure 4). No significant differences were found between game formats in the RPEs and RPFs for girls (*p* > 0.05) (Table 1).

### 3.3. Technical Analysis

For the total number of shots, more shots occurred in S3 + 1 and S4 compared to M5 and L5 (*p* < 0.05; ES = 0.8 to 1.1). In contrast, no differences were observed for goals, successful shots, or duels in all the formats. For girls, the total amount of goals was higher in S3 + 1 than in M4, M5, and L5 (*p* < 0.05; ES = 0.9 to 1.3), as well as in S4 compared to M5 (*p* = 0.029; ES = 1.3 (0.3; 1.6)). In addition, situation S3 + 1 had more shots than M5 and L5 (*p* < 0.05; ES = 1.0 to 1.6). Furthermore, girls were less successful with shots in M5 than in S3 + 1 and S4 (*p* < 0.05; ES = 0.9 to 1.6) (Table 2).

### 3.4. Gender

The boys covered more TD in S3 + 1, S4, M5, and L5 compared to the girls (*p* < 0.05; ES = 0.8 to 0.9). Furthermore, the boys reached higher *V*_peak_ during S4 and M5 compared to the girls (*p* < 0.05; ES = 0.7 to 0.9). Moderate higher sprints were observed in the M4 format for boys compared to girls (*p* = 0.020; ES = 0.7 [0.2; 1.3]). Notably, the jog distance was higher for boys during S3 + 1, S4, M4, M5, and L5 compared to girls (*p* < 0.05; ES = 0.6 to 1.2). In addition, sprinting in M4 and L5 was higher for boys than girls (*p* < 0.05; ES = 0.8 to 0.5). Moreover, Acc_total_ and Dec_total_ were higher in S3 + 1 for boys compared to girls (*p* < 0.05; ES = 0.7 to 1.0). Acc_<1.5_ was moderately higher during S4 in boys compared to girls (*p* = 0.044; ES = 0.6 (0.1; 1.2)). Notably, boys had higher numbers of decelerations during L5 compared to girls in Dec_>2.3_ (*p* = 0.021; ES = 0.6 (0.2; 1.1)). Furthermore, S3 + 1 and S4 formats had more decelerations for boys compared to girls (*p* < 0.05; ES = 0.8 to 1.2). Conversely, in Dec_1.5-2.3_, girls had more decelerations in M4 than boys (*p* = 0.009; ES = 0.9 (1.5; 0.4)).

**Figure 5:**
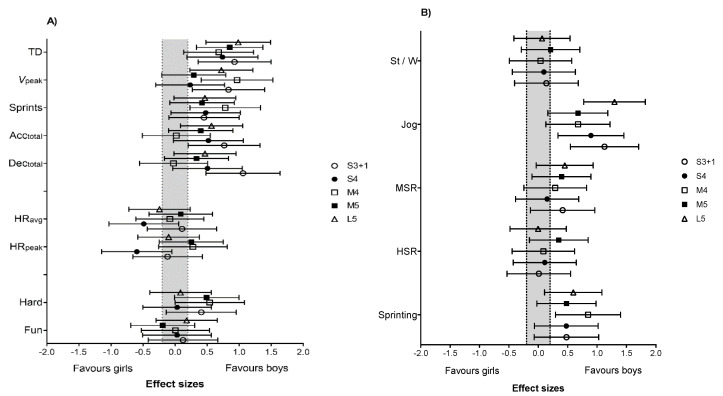

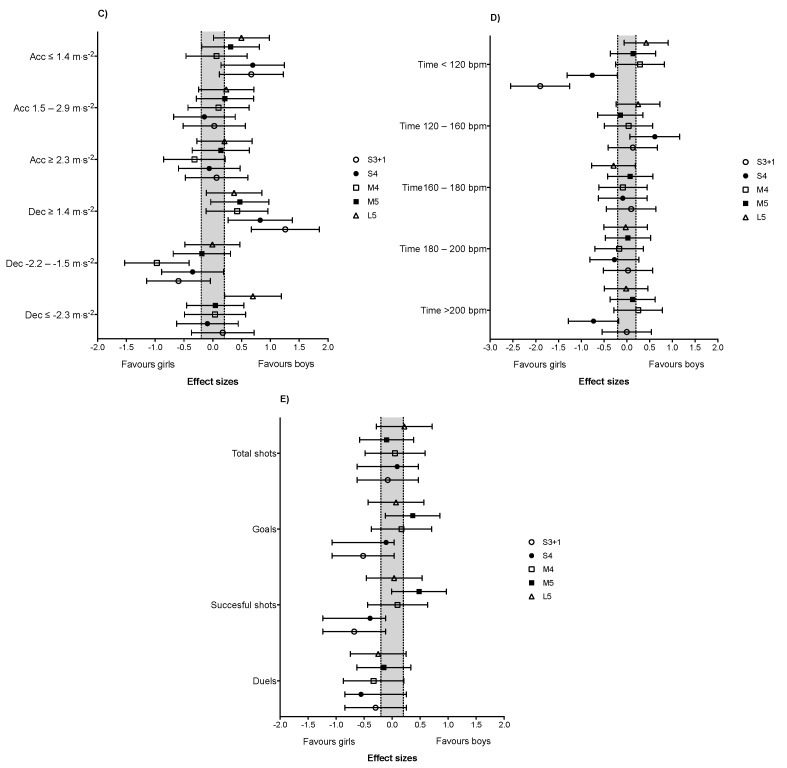
Differences in (**A**) overall physical and physiological demands, (**B**) activity profile, (**C**) accelerations and decelerations, (**D**) heart rate, and (**E**) technical demands between boys and girls during different U9 handball games. The forest plots are effect sizes (90% CI). S3 + 1: small size, 3 v 3 + offensive goalkeeper; S4: small size, 4 v 4; M4: medium size, 4 v 4; M5: medium size, 5 v 5; L5: large size, 5 v 5. St/W: standing/walking; MSR: moderate-speed running; HSR: high-speed running.

The girls had higher Time_<120_ in S3 + 1 and S4 compared to the boys (*p* < 0.05; ES = 0.7 to 1.9) (Table 1). In addition, Time_>200_ in S4 was moderately higher for girls compared to boys (*p* = 0.034; ES = 0.7 (1.2; 0.1)). A detailed representation of the differences in activity profile, heart rate, subjective ratings, and technical involvement is reported in Figure 5. 

## 4. Discussion

This study provides the first detailed analysis of movement patterns and heart rates in U9 team handball for boys and girls, showing that the exercise intensity, heart rates, and technical involvement are high during small, medium, and large-sized games in all investigated formats. When comparing game formats, we observed higher distances covered and more sprints with L5 but a lower number of accelerations and decelerations compared to all the other formats. Notably, heart rates were similar between game formats. Irrespective of game format, boys covered 977–1320 m and girls covered 846–1124 m. For boys and girls, remarkably in the L5 format, TD, *V*_peak_, sprints, HSR, and sprinting were higher, whereas St/W, JOG, Acc_total_, Dec_total_, Acc_<1.5_, Acc_1.5–2.3_, Acc_>1.5_, Dec_<1.5,_ Dec_1.5–2.3_, and Dec_>1.5_ were lower than other formats and, on many occasions, significantly different. This may be because there is more room for sprinting and high-intensity running on larger pitches, which is supported by the greater distance covered with high-intensity running and higher *V*_peak_ during games on larger pitch sizes (40 × 20 m) compared with small pitches (20 × 13 m) in adult football players [25]. Interestingly, no differences were found between S4 and M4 in any variables (physical, physiological, subjective perception, and technical). As we already reported, other team sports showed that manipulating the player numbers and the pitch size can alter the exercise intensity (i.e., distance covered, jogging and walking, heart rate, and tackling, dribbling, goal attempts, and passes) during a game in different sports [8]. The forces generated while rapidly changing direction, stopping, and landing, as well as during jumping and shooting, may confer excellent osteogenic properties to team handball [26]. It is well known from cross-sectional studies that participation in sports activities is associated with markedly higher muscle mass and bone mineralization, as well as better coordination and postural balance [27,28], and a longitudinal intervention study with 8–10-year-old children has shown that participation in school-based small-sized ball games enhances the same parameters [29]. The mean HR was high for boys and girls, at 166–176 bpm and 165–175 bpm, respectively, in all game formats. A high HR during sports and, specifically, team handball match-play, irrespective of game format and gender, is important for the health profile of children [30]. Aerobic high-intensity training (>90% maximum HR) has been shown to be superior to moderate continuous training in improving cardiorespiratory fitness [31,32], which has been identified as a strong independent predictor of the risk of cardiovascular diseases and mortality [33]. Sports participation is an effective way to improve aerobic and anaerobic fitness, especially participation in high-intensity ball games [34]. For the Time_180–200_ and Time_>200_ in S3 + 1 format, young girl and boy team handball players spent more time above 180 bpm, which is not significantly different but working at a high intensity for more time could improve cardiorespiratory fitness positively [35]. No differences occurred in subjective perception between different game formats, in contrast with other studies [6,36] that found that larger courts felt more physically demanding. In our study, we had more goals and more shots in the small size pitch (S3 + 1, S4), as was also observed in a study by Randers and colleagues [7], where smaller pitches created more technical actions and may seem logical, as ball contacts are higher during a game with fewer players [37]. Interestingly, no differences were found in 1 v 1 duels in all the formats, that the players may try to score or shot faster in games with small size pitches. Involvement with many relevant activities is important in terms of motivation for children [38], as it helps the players to continue as active handball players. Maturation at this stage is still early, whereas it seems that the physiological load of the game is higher for boys than for girls, with many differences between them, as is supported by the work of Michalsik and colleagues [3] in the different distance zones, except for the TD, which females covered more of. A possible explanation is that boys have more self-confidence and perceived self-competence, making the game more demanding [39]. Only one significant difference was observed in favor of the girls in Dec_1.5–2.3_, which had more decelerations in the M4 format. However, for physical loading between sexes, similar HR values were found, with only three comparisons, girls spent more time below 120 bpm in S3 + 1 and S4 compared to boys for Time_>200_ in S4. Additionally, no significant differences were found for subjective perceptions or the technical analysis. In conclusion, having both genders mixed in the same format and game would possibly be very demanding for girls in terms of activity patterns at this age.

It is important to underline some limitations inherent to this study, Firstly, physical and physiological demands were compared across game formats of various pitch sizes and numbers of players, and thus, relative space per player was not constant. Secondly, maximum HR, maximal aerobic speed, and maximal sprinting speed were not assessed. The use of fixed HR and speed zones does not reflect the actual individual capacity, possibly resulting in under- or overestimating the real physical and physiological demands of the game. Although the technical analysis was carried out by experienced handball coaches, this analysis could be somewhat subjective. Thus, our technical analysis should be interpreted with caution. Finally, for logistical reasons, we were unable to describe the physical levels of the players. Future studies are warranted to use individualized HR and speed zones to accurately quantify the physical and physiological demands of youth team handball as well as physical evaluations of the players. In this context, the fitness component of max speed can be adopted in future studies as suggested by [40].

## 5. Conclusions

In summary, the HR and high-intensity distances are high in U9 team handball matches irrespective of the game format. The present data provide insight into how different game formats influence the physiological and the physical loading and evidence that various types of match-plays can contribute significantly to the improvement in the musculoskeletal and cardiovascular fitness of U9 boys because of high HRs and high-intensity running distances, along with multiple accelerations and specific actions with considerable impact. In all the game formats, physical loading seems similar but, interestingly, on the large pitch, the physiological load was higher. Playing with fewer players on smaller pitches resulted in minor changes to the physiological loading but elevated the technical involvement of players, which favors the use of smaller formats to emphasize technical demands. Several differences between girls and boys were found in U9 team handball players that should be considered when planning games for boys and girls separately or for mixed-gender games. The various game types could provide valuable information to coaches in the selection of players or training guidance. We would recommend the use of games with fewer players on smaller courts for U9 boys and girls since we believe that technical development is the most important factor at this age.

## Figures and Tables

**Table 1:** Differences in peak and average values and total distance between game formats.

Variables (U9)	Sex	S3 + 1	S4	M4	M5	L5
Activity profile						
TD (m)	Boys	1133 ± 171	988 ± 141	1106 ± 157	977 ± 172 ^a^	1320 ± 232 ^a,b,c,d^
	Girls	965 ± 195	878 ± 159	999 ± 156	846 ± 124 ^c^	1125 ± 134 ^a,b,d^
V_peak_ (km·h^−1^)	Boys	21.4 ± 3.2	20.1 ± 3.0	22.4 ± 3.1	20.7 ± 2.8	23.6 ± 3.0 ^b,d^
	Girls	19.0 ± 2.3	19.5 ± 2.6	19.5 ± 2.8	19.9 ± 2.7	21.6 ± 2.4 ^a^
Sprints (counts)	Boys	5.1 ± 5.4	2.9 ± 3.6	5.8 ± 5.2	4.6 ± 5.5	11.9 ± 7.5 ^a,b,c,d^
	Girls	2.9 ± 3.5	1.5 ± 1.5	2.4 ± 2.6	2.7 ± 2.5	8.4 ± 7.6 ^a,b,c,d^
Acc_total_ (counts)	Boys	212.0 ± 20.3	209.0 ± 21.2	200.7 ± 20.0	186.1 ± 21.6 ^a,b^	172.2 ± 23.2 ^a,b,c,d^
	Girls	198.0 ± 14.4	197.1 ± 25.3	200.3 ± 21.4	177.4 ± 21.7 ^a,b,c^	160.7 ± 15.0 ^a,b,c,d^
Dec_total_ (counts)	Boys	219.8 ± 17.3	209.8 ± 21.7	203.6 ± 17.8	191.5 ± 19.7 ^a,b,c^	182.7 ± 24.0 ^a,b,d^
	Girls	201.1 ± 18.1	197.1 ± 26.4	204.1 ± 17.8	184.7 ± 20.9 ^a,b,c^	173.2 ± 13.6 ^a,b,d^
Heart rate						
HR_avg_ (bpm)	Boys	175.8 ± 10.3	165.9 ± 11.2	167.9 ± 11.5	165.8 ± 13.1	169.7 ± 14.8
	Girls	174.7 ± 10.2	171.1 ± 9.7	168.8 ± 11.1	164.6 ± 13.5	172.8 ± 8.7
HR_peak_ (bpm)	Boys	195.1 ± 10.3	185.6 ± 10.1 ^a^	192.3 ± 10.5	189.2 ± 11.6	191.2 ± 11.1
	Girls	196.3 ± 8.6	191.9 ± 11.4	189.4 ± 9.5	186.1 ± 12.8	192.3 ± 8.3
Subjective perceptions						
RPE (AU)	Boys	8.4 ± 3.8	10.2 ± 4.2	8.5 ± 4.1	9.2 ± 5.2	6.9 ± 4.9
	Girls	6.9 ± 3.3	10.1 ± 3.0	6.4 ± 3.3	6.8 ± 4.4	6.5 ± 4.8
RPF (AU)	Boys	4.3 ± 4.6	5.2 ± 4.7	5.2 ± 4.3	4.5 ± 5.1	5.0 ± 5.4
	Girls	3.8 ± 2.7	5.0 ± 3.7	5.1 ± 2.8	5.4 ± 3.6	4.1 ± 4.7

Data are mean ± SD. S3 + 1: small size, 3 vs. 3 + offensive goalkeeper; S4: small size, 4 vs. 4; M4: medium size, 4 vs. 4; M5: medium size, 5 vs. 5; L5: large size, 5 vs. 5. Acc_total_: total accelerations; Dec_total_: total decelerations; HR_avg_: average heart rate; HR_peak_: peak heart rate; TD: total distance; V_peak_: peak speed attained; RPE: rating of perceived exertion; RPF: rating of perceived enjoyment/fun. ^a^ denotes significant differences compared to S3 + 1; ^b^ to S4; ^c^ to M4; ^d^ to M5 (*p* ≤ 0.05).

**Table 2:** Differences in technical demands between game formats. Data are mean ± *SD*.

Variables	Sex	S3 + 1	S4	M4	M5	L5
Shots (counts)	Boys	7.0 ± 4.1	6.4 ± 3.9	4.9 ± 3.3	3.5 ± 2.6 ^a,b^	3.2 ± 2.1 ^a,b^
	Girls	7.4 ± 3.5	6.0 ± 5.2	4.7 ± 2.8	3.8 ± 3.0 ^a^	2.8 ± 2.0 ^a^
Goals (counts)	Boys	2.5 ± 2.4	2.2 ± 2.1	2.0 ± 2.0	1.1 ± 1.3	1.1 ± 1.0
	Girls	3.9 ± 2.9	2.4 ± 1.5	1.6 ± 1.4 ^a^	0.7 ± 0.9 ^b^	1.0 ± 1.3 ^a^
Successful shots (counts)	Boys	35.2 ± 29.2	34.7 ± 23.9	38.0 ± 30.1	29.2 ± 32.0	32.2 ± 28.9
	Girls	53.7 ± 23.9	47.4 ± 41.3	35.3 ± 25.8	15.4 ± 22.5 ^a,b^	31.0 ± 38.9
Duels (counts)	Boys	1.1 ± 1.2	0.7 ± 1.3	0.7 ± 1.1	0.5 ± 0.9	0.4 ± 0.6
	Girls	1.7 ± 2.8	1.6 ± 2.1	1.2 ± 1.7	0.6 ± 1.0	0.6 ± 1.2

Data are mean ± SD. S3 + 1: small size, 3 vs. 3 + offensive goalkeeper; S4: small size, 4 vs. 4; M4: medium size, 4 vs. 4; M5: medium size, 5 vs. 5; L5: large size, 5 vs. 5. ^a^ denotes significant differences compared to S3 + 1; ^b^ to S4.

## References

[1] Gorostiaga E.M., Granados C., Ibanez J., Izquierdo M. (2005). Differences in physical fitness and throwing velocity among elite and amateur male handball players. Int. J. Sports Med..

[2] Povoas S.C., Seabra A.F., Ascensao A.A., Magalhaes J., Soares J.M., Rebelo A.N. (2012). Physical and physiological demands of elite team handball. J. Strength Cond. Res..

[3] Michalsik L.B., Madsen K., Aagaard P. (2014). Match performance and physiological capacity of female elite team handball players. Int. J. Sports Med..

[4] Chelly M.S., Hermassi S., Aouadi R., Khalifa R., Van den Tillaar R., Chamari K., Shephard R.J. (2011). Match analysis of elite adolescent team handball players. J. Strength Cond. Res..

[5] Souhail H., Castagna C., Mohamed H.Y., Younes H., Chamari K. (2010). Direct validity of the yo-yo intermittent recovery test in young team handball players. J. Strength Cond. Res..

[6] Madsen M., Ermidis G., Rago V., Surrow K., Vigh-Larsen J.F., Randers M.B., Krustrup P., Larsen M.N. (2019). Activity Profile, Heart Rate, Technical Involvement, and Perceived Intensity and Fun in U13 Male and Female Team Handball Players: Effect of Game Format. Sports.

[7] Randers M.B., Andersen T.B., Rasmussen L.S., Larsen M.N., Krustrup P. (2014). Effect of game format on heart rate, activity profile, and player involvement in elite and recreational youth players. Scand. J. Med. Sci. Sports.

[8] Halouani J., Chtourou H., Gabbett T., Chaouachi A., Chamari K. (2014). Small-sided games in team sports training: A brief review. J. Strength Cond. Res..

[9] Aroso J., Rebelo A., Gomes-Pereira J. (2004). Physiological impact of selected game-related exercises. J. Sports Sci..

[10] Owen A.L., Wong del P., McKenna M., Dellal A. (2011). Heart rate responses and technical comparison between small- vs. large-sided games in elite professional soccer. J. Strength Cond. Res..

[11] Malina R.M., Eisenmann J.C., Cumming S.P., Ribeiro B., Aroso J. (2004). Maturity-associated variation in the growth and functional capacities of youth football (soccer) players 13–15 years. Eur. J. Appl. Physiol..

[12] Malina R.M.C., Bar-Or O. (2004). Growth, Maturation, and Physical Activity.

[13] Hill-Haas S.V., Rowsell G.J., Dawson B.T., Coutts A.J. (2009). Acute physiological responses and time-motion characteristics of two small-sided training regimes in youth soccer players. J. Strength Cond. Res..

[14] Barbero-Alvarez J.C., Gomez-Lopez M., Castagna C., Barbero-Alvarez V., Romero D.V., Blanchfield A.W., Nakamura F.Y. (2017). Game Demands of Seven-A-Side Soccer in Young Players. J. Strength Cond. Res..

[15] Sanchez-Sanchez J., Sanchez M., Hernandez D., Ramirez-Campillo R., Martinez C., Nakamura F.Y. (2017). Fatigue In U12 Soccer-7 Players During Repeated One-Day Tournament Games—A Pilot Study. J. Strength Cond. Res..

[16] Akyildiz Z., Yildiz M., Clemente F.M. (2020). The reliability and accuracy of Polar Team Pro GPS units. Proc. Inst. Mech. Eng. Part P J. Sports Eng. Technol..

[17] Rebelo A., Brito J., Seabra A., Oliveira J., Drust B., Krustrup P. (2012). A new tool to measure training load in soccer training and match play. Int. J. Sports Med..

[18] Wewers M.E., Lowe N.K. (1990). A critical review of visual analogue scales in the measurement of clinical phenomena. Res. Nurs. Health.

[19] Liu H., Gomez M.-Á., Lago-Peñas C., Sampaio J. (2015). Match statistics related to winning in the group stage of 2014 Brazil FIFA World Cup. J. Sports Sci..

[20] Michalsik L.B., Madsen K., Aagaard P. (2015). Technical match characteristics and influence of body anthropometry on playing performance in male elite team handball. J. Strength Cond. Res..

[21] Cnaan A., Laird N.M., Slasor P. (1997). Using the general linear mixed model to analyse unbalanced repeated measures and longitudinal data. Stat. Med..

[22] Hopkins W.G., Marshall S.W., Batterham A.M., Hanin J. (2009). Progressive statistics for studies in sports medicine and exercise science. Med. Sci. Sports Exerc..

[23] Batterham A.M., Hopkins W.G. (2006). Making meaningful inferences about magnitudes. Int. J. Sports Physiol. Perform..

[24] Hopkins W. (2007). A spreadsheet for deriving a confidence interval, mechanistic inference and clinical inference from a P value. Sportscience.

[25] Casamichana D., Castellano J. (2010). Time-motion, heart rate, perceptual and motor behaviour demands in small-sides soccer games: Effects of pitch size. J. Sports Sci.

[26] Missawi K., Zouch M., Chakroun Y., Chaari H., Tabka Z., Bouajina E. (2016). Handball Practice Enhances Bone Mass in Specific Sites Among Prepubescent Boys. J. Clin. Densitom..

[27] Seabra A., Marques E., Brito J., Krustrup P., Abreu S., Oliveira J., Rego C., Mota J., Rebelo A. (2012). Muscle strength and soccer practice as major determinants of bone mineral density in adolescents. Jt. Bone Spine.

[28] Vicente-Rodriguez G., Ara I., Perez-Gomez J., Dorado C., Calbet J.A. (2005). Muscular development and physical activity as major determinants of femoral bone mass acquisition during growth. Br. J. Sports Med..

[29] Larsen M.N., Nielsen C.M., Helge E.W., Madsen M., Manniche V., Hansen L., Hansen P.R., Bangsbo J., Krustrup P. (2018). Positive effects on bone mineralisation and muscular fitness after 10 months of intense school-based physical training for children aged 8–10 years: The FIT FIRST randomised controlled trial. Br. J. Sports Med..

[30] Larsen M.N., Nielsen C.M., Madsen M., Manniche V., Hansen L., Bangsbo J., Krustrup P., Hansen P.R. (2018). Cardiovascular adaptations after 10 months of intense school-based physical training for 8- to 10-year-old children. Scand. J. Med. Sci. Sports.

[31] Helgerud J., Hoydal K., Wang E., Karlsen T., Berg P., Bjerkaas M., Simonsen T., Helgesen C., Hjorth N., Bach R. (2007). Aerobic high-intensity intervals improve VO2max more than moderate training. Med. Sci. Sports Exerc..

[32] Nybo L., Sundstrup E., Jakobsen M.D., Mohr M., Hornstrup T., Simonsen L., Bulow J., Randers M.B., Nielsen J.J., Aagaard P. (2010). High-intensity training versus traditional exercise interventions for promoting health. Med. Sci. Sports Exerc..

[33] Archer E., Blair S.N. (2011). Physical activity and the prevention of cardiovascular disease: From evolution to epidemiology. Prog. Cardiovasc. Dis..

[34] Bendiksen M., Williams C.A., Hornstrup T., Clausen H., Kloppenborg J., Shumikhin D., Brito J., Horton J., Barene S., Jackman S.R. (2014). Heart rate response and fitness effects of various types of physical education for 8- to 9-year-old schoolchildren. Eur. J. Sport Sci..

[35] Sperlich B., Zinner C., Heilemann I., Kjendlie P.L., Holmberg H.C., Mester J. (2010). High-intensity interval training improves VO(2peak), maximal lactate accumulation, time trial and competition performance in 9–11-year-old swimmers. Eur. J. Appl. Physiol..

[36] Corvino M., Tessitore A., Minganti C., Sibila M. (2014). Effect of Court Dimensions on Players’ External and Internal Load during Small-Sided Handball Games. J. Sports Sci. Med..

[37] Abrantes C.I., Nunes M.I., Maçãs V.M., Leite N.M., Sampaio J.E. (2012). Effects of the number of players and game type constraints on heart rate, rating of perceived exertion, and technical actions of small-sided soccer games. J. Strength Cond. Res..

[38] Bangsbo J., Krustrup P., Duda J., Hillman C., Andersen L.B., Weiss M., Williams C.A., Lintunen T., Green K., Hansen P.R. (2016). The Copenhagen Consensus Conference 2016: Children, youth, and physical activity in schools and during leisure time. Br. J. Sports Med..

[39] O’Connor D., Gardner L., Larkin P., Pope A., Williams A.M. (2020). Positive youth development and gender differences in high performance sport. J. Sports Sci.

[40] Mendez-Villanueva A., Buchheit M., Simpson B., Bourdon P.C. (2013). Match play intensity distribution in youth soccer. Int. J. Sports Med..

